# Supplementary material for: Intramolecular dynamic coupling slows surface relaxation of polymer glasses
Source: Nat Commun. 2024 Jul 19;15:6082. doi: 10.1038/s41467-024-50398-7 (PMC11271542; doi:10.1038/s41467-024-50398-7)
Supplement: Supplementary file 1 — Supplementary Information [file 41467_2024_50398_MOESM1_ESM.pdf]

Supplementary Information for:

Intramolecular Dynamic Coupling Slows Surface Relaxation of Polymer Glasses

Houkuan Tian,[1,#] Jintian Luo,[1,#] Qiyun Tang,[2,*] Hao Zha,[1] Rodney D. Priestley,[3,*] Wenbing Hu[4] and Biao Zuo[1,5,6*]

[1] School of Chemistry and Chemical Engineering, Key Laboratory of Surface & Interface Science of Polymer Materials of Zhejiang Province, Zhejiang Sci-Tech University, Hangzhou 310018, China

[2] Key Laboratory of Quantum Materials and Devices of Ministry of Education, School of Physics, Southeast University, Nanjing 211189, China

[3] Department of Chemical and Biological Engineering, Princeton Institute for the Science and Technology of Materials, Princeton University, Princeton, New Jersey 08544, USA

[4] Department of Polymer Science, School of Chemistry and Chemical Engineering, State Key Lab of Coordination Chemistry, Nanjing University, Nanjing 210023, China

[5] Zhejiang Provincial Innovation Center of Advanced Textile Technology, Shaoxing 312000, China

[6] Zhejiang Sci-Tech University Shengzhou Innovation Research Institute, Shengzhou 312400, China

* Corresponding author: qtang@seu.edu.cn for Q. T.; rpriestl@princeton.edu for R. D. P.; chemizuo@zstu.edu.cn for B. Z.

# The authors contributed equally to this work.

1. Physical characteristics of polymers used in the study

Supplementary Table 1. Physical characteristics of polymers

Sample [a]	f_{PFS} (mol %)	M_w (kDa)	PDI	T_g [b]	Surface free energy [c] (mN/m)
PPFS	100.0	85	1.18	373 K	20.3
PMMA	0.0	88	1.19	390 K	45.7
PMMA-*sta*-PPFS-1	2.2	86	1.21	389 K	44.5
PMMA-*sta*-PPFS-2	5.2	86	1.21	388 K	42.5
PMMA-*sta*-PPFS-3	7.7	86	1.21	387 K	40.6
PMMA-*sta*-PPFS-4	10.6	87	1.18	387 K	38.6

a: P(MMA-*sta*-PFS) was synthesized using atom transfer radical polymerization. The nearly identical reactivity ratios of MMA and PFS ensure the statistical distribution of PFS in the copolymers. b: The bulk glass transition temperature (T_g) was determined by differential scanning calorimetry (DSC) using a cooling rate of 10 K/min. c: The surface free energy was calculated based on the contact angles of water and diiodomethane (see Supplementary Table 2 for the corresponding surface tension values) using Owens and Wendt's theory (see Supplementary Eq.1) [1].

Owens and Wendt's theory:

$$(\gamma_s^D \gamma_l^D)^{1/2} + (\gamma_s^P \gamma_l^P)^{1/2} = \gamma_l (\cos\theta + 1)/2 \qquad \text{(Supplementary Eq. 1)}$$

γ represent the surface tension values; the subscripts s and l denote the solid film and testing liquid; superscripts p and D represent polar and non-polar components of the surface tension. θ is the contact angle of liquid on the film surfaces.

Supplementary Table 2. The polar and non-polar surface tension parameters of the testing liquids

Testing liquids	γ_l (mJ/m^2)	γ_l^P (mJ/m^2)	γ_l^D (mJ/m^2)
water	72.8	51.0	21.8
diiodomethane	50.8	0.0	50.8

2. Synthesis of the copolymers of P(MMA-*sta*-PFS)

2.1 Materials

Methyl methacrylate (MMA) and ethyl 2-bromoisobutyrate (BiEB) were purchased from Shanghai Macklin Biochemical Co., Ltd.; Pentafluorostyrene (PFS) was provided by J&K Scientific Co., Ltd.; CuBr and *N*,*N*,*N'*,*N''*,*N''*-Pentamethyldiethylenetriamine (PMDETA) was from Shanghai Aladdin Bio-Chem Technology Co., Ltd..

2.2 Synthesis of P(MMA-sta-PFS) and homo-PMMA

According to ref. [2], P(MMA-*sta*-FS) were synthesized by means of atom transfer radical polymerization (ATRP) using BiEB as initiator, CuBr as catalyst, and PMDETA as ligand. The synthesis route was shown in Supplementary Figure 1. Briefly, the comonomers of MMA and PFS were firstly purified by chromatography to remove polymerization inhibitors and then co-dissolved in trifluorotoluene. The synthesis was conducted with the sequential addition of BiEB, CuBr and PMDETA in a closed flask under the protection of N_2 at 373 K. The residual CuBr in the reaction mixture was removed by chromatography with an alkaline alumina column. The product was precipitated in methanol as white solid and dried under vacuum at 60 °C for 24h to remove any residual organic solvent. The homo-PMMA was polymerized with the same process and synthesis conditions. The chemical structure of the synthesized polymer was characterized by ^{1}H-NMR spectroscopy (Supplementary Figure 2) and the molar fraction of PFS in copolymer was determined by the fluorine element analysis using the ignition method [3]. The molecular weight and molecular wight distribution were determined by gel permeation chromatography (GPC) using a Waters 515 GPC apparatus with THF as eluent at a flow rate of 0.5 ml/min.

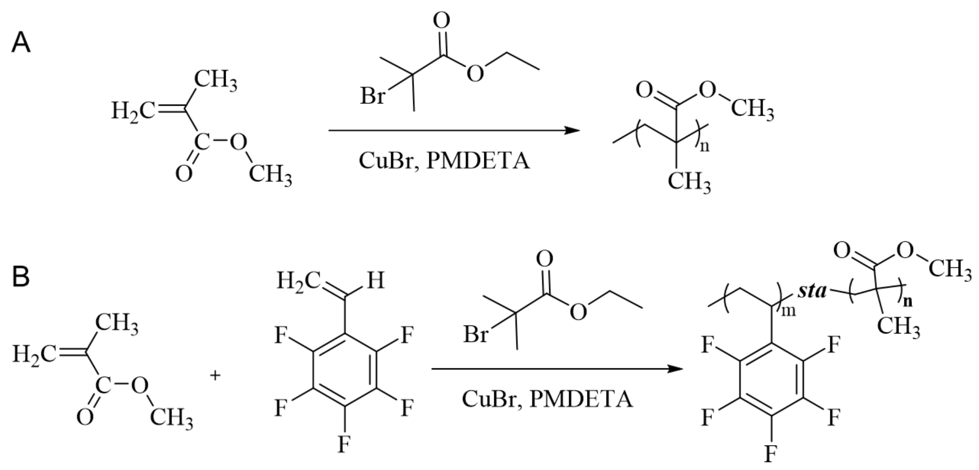

Supplementary Figure 1. Synthesis of PMMA and P(MMA-*sta*-FS). Synthesis route of (a) homo-PMMA and (b) P(MMA-*sta*-FS).

It is noteworthy that the reactivity ratios of MMA (r_2) and PFS (r_1) were 0.88 and 1.17, respectively; see the details of measurement in section below. The reactivity ratios of PFS and MMA are close to 1, indicating the nearly identical preference of the propagating radical to the either monomer which produces a perfectly random incorporation of both types of monomers in the chains, *i.e.*, a statistical copolymer of P(MMA-*sta*-PFS). This allows to prepare the surface loops with statistically identical sizes.

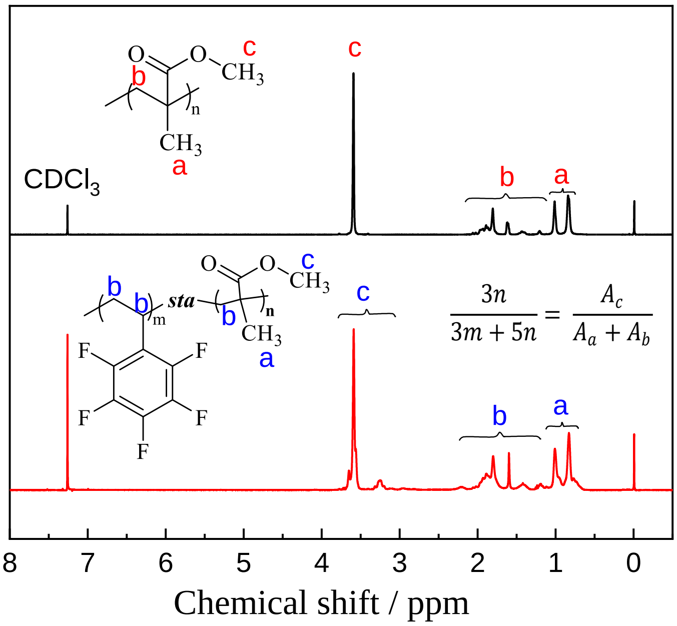

Supplementary Figure 2. Chemical structure of the synthesized polymers. The ^{1}H-NMR spectra of the synthesized PMMA and P(MMA-*sta*-PFS) using deuterated chloroform as solvent.

2.3 Determination of reactivity ratios

The reactivity ratios of MMA (r_1) and PFS (r_2) were estimated by the Fineman-Ross method [4]. Briefly, copolymers with a series of comonomer feeding ratios were synthesized by ATRP under identical conditions, and the conversions were kept lower than 20%. At prescribed time points, the copolymer and comonomer compositions in the reaction mixture were determined by ^{1}H-NMR spectroscopy, and can be related to Supplementary Eq. 2:

$$\frac{f-1}{F} = -r_2\frac{f}{F^2} + r_1 \qquad \text{(Supplementary Eq. 2)}$$

Here, f represents the molar ratio of MMA in the residual comonomers, F represents the molar ratio of MMA sequences in the synthesized copolymer. The reactivity ratios of MMA (r_2) and PFS (r_1) can be extracted from the linear relation between $(f\text{-}1)/F$ and f/F^2 (see Supplementary Figure 3). The estimated value of r_1 and r_2 were 0.88 and 1.17, respectively, close to the values (*i.e.*, r_1 = 0.98, r_2 =

0.90) reported by Pryor *et al.* [5]

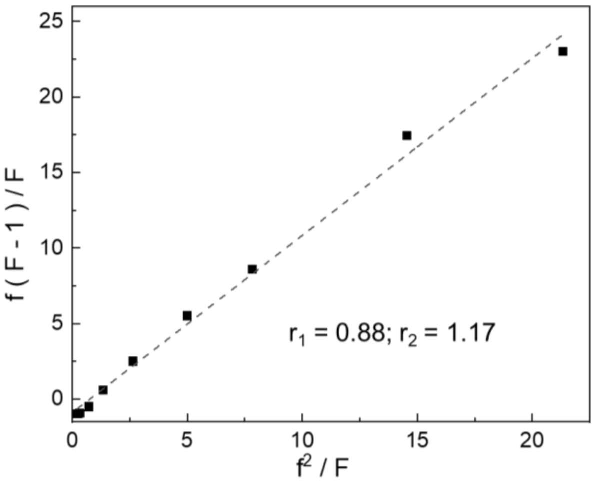

Supplementary Figure 3. The reactivity ratios of MMA and PFS monomers. The relation between (f-1)/F and f/F^2. The dashed line represents the best linear fit of Supplementary Eq. 2.

The monomer reactivity ratios of MMA and PFS are close to each other with $r_1 \times r_2 = 1.03$, meaning that the propagating radical has the similar preference for MMA and PFS and therefore produces a perfectly random incorporation of both types of monomers. Therefore, the obtained copolymer of P(MMA-*sta*-PFS) is a real random copolymer with MMA and PFS arranged in a nearly perfectly random manner along the chain. This allows us to prepare the surface loop with statistically identical loop height.

3. Film preparation

Native oxide layer covered (SiO_x) and hydrogen terminated (Si-H) silicon wafers were used as substrates for the films. The detailed procedure of substrate preparation was depicted in ref [6]. The RMS roughness of the cleaned substrate surface was below 0.5 nm examined by AFM. P(MMA-*sta*-FS) copolymer and PMMA homopolymer were co-dissolved in trifluorotoluene at prescribed molar ratios. The mixture solution was filtered through a PTFE membrane filter (0.22 μm) and then spin-casted onto cleaned substrates. Film thickness was controlled by adjusting the total concentration. Resultant films were annealed at T_g - 20 K for 24 hrs under vacuum to remove residual solvent and then annealed above T_g (T_g + 35 K) to expediate segregation of the PFS units to toward the air interface.

4. Characterizations of surface structure of the films

We used the water contact angle, X-ray photoelectron spectroscopy (XPS) and sum-frequency generation (SFG) spectrometer to measure the surface structure of the blend films. The water contact angle of the films was measured using the sessile drop method at room temperature with a Krüss DSA-10 contact angle goniometer (Hamburg, Germany). The volumes of the water droplets used were 5 μL. The reported contact angle values were the averages of eight measurements taken within 10 s of applying each drop of liquid. XPS was performed using a PHI5000C ESCA system with an Al Kα X-ray source (1253.6 eV) to investigate the surface F/C atomic ratio. The takeoff angle of the X-ray was 15° corresponding to an analytical depth of 2.3 nm. A commercial SFG spectrometer (EKSPLA, Lithuania) was used to examine the surface structure of the films. In the measurement, 532 nm visible and tunable infrared laser beams overlapped at the surface of the films to generate sum frequency signals. The incident angles of visible and IR laser were 60° and 55°, respectively. SFG spectra with *ssp* (SF output, visible input, and infrared input) and *ppp* polarization combinations were collected.

5. Validation of the formation of loop structure by surface compositional analysis

Supplementary Figure 4A illustrates schematically the formation of loop structure at surface driven by surface adsorption of PFS and bulk segregation of MMA. In addition to the snapshot of a simulated surface-adsorbed copolymer chain shown in the inset of Figure 1B, characterizations of the surface structure and properties of the PMMA/P(MMA-*sta*-PFS) films by water contact angle measurements, XPS and SFG spectroscopy confirm this picture. Both the water contact angle and surface F/C atomic ratio increase with prolonged annealing, indicating surface adsorption of PFS (Supplementary Figure 4B); concurrently, the intensity of the symmetrical C-H stretching of $-OCH_3$ in MMA (i.e., $-OCH_3$ s) decreases in the SFG spectra, reflecting the bulk segregation of MMA (Supplementary Figure 4C).

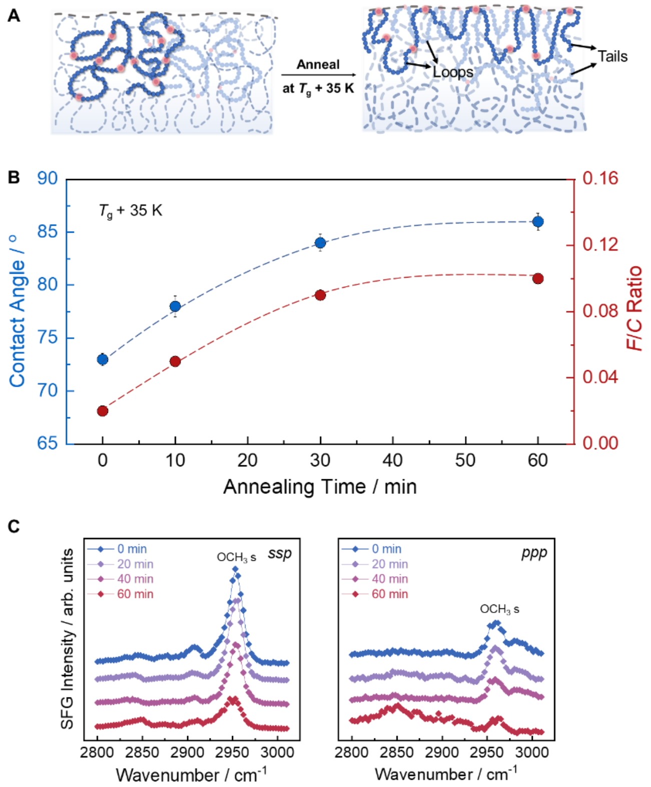

Supplementary Figure 4. Experimental evidences for the formation of loops at the surface of blended films. (A) Scheme for the formation of surface loops during annealing. (B) Water contact angles, surface F/C ratios obtained by XPS and (C) SFG spectra with *ssp* and *ppp* polarization combinations of the PMMA/P(MMA-*sta*-PFS) films (f_{PFS} = 0.104, X_c = 15 %) annealed at 423 K for various times. Although the SFG spectra and water contact angle were affected by both the concentration and orientation of PFS at surface, the consistency of the results with compositional analysis by XPS indicates the negligible influence of orientation changes and evidences surface segregation of PFS and depletion of MMA from the surface during annealing. The takeoff angle of photoelectrons in the XPS measurement is 15°, corresponding to a sampling depth of 2.3 nm. Thickness of the films (h) is 300 nm. Error bars are ± standard errors.

6. Monte Carlo simulation of the polymer films

In our coarse-grained model, we choose 4 repeat units of PMMA as a monomer, which generates the coarse-grained monomer size of $1.0\ nm$, closing to that of the Kuhn segment. With this coarse-grained procedure, the PMMA is modelled as a polymer chain containing 220 monomers. The PFS monomers take similar coarse-grained procedure. The P(MMA-*sta*-PFS) with distinct f_{PFS} has molecular weight around $86 \sim 87$ kDa. This gives the total monomer of P(MMA-*sta*-PFS) around $N \approx 200$. In our model, we set the chain length to a fixed value of $N = 200$, and the number of PFS monomer takes the value of 4, 10, 16, and 22. This yields the mole fractions of f_{PFS} in simulations with 0.02, 0.05, 0.08, 0.11, which are consistent with experimental parameters shown in Supplementary Table 1. Here we ignore the slight volume difference between the PMMA and PFS monomers, and set each monomer size as $b_0 = 1.0\ nm$.

We use the lattice model [7,8] of polymers chains to study the mobility of P(MMA-*sta*-PFS) thin films. This model combines the kink-jump methods and the slithering diffusion terminated by extending the nearest kink conformation along the chain, originally proposed by de Gennes [9,10,11]. This model was proven to be a powerful technique in the simulations of polymer systems, which have been successfully employed to study the physical aging of polymer thin films [7] and even the surface crystallization in polymer solutions [8]. Here we choose a system of $40 \times 40 \times 250$ grid sites, with the periodic conditions in x, y, and z axis. As each monomer of P(MMA-*sta*-PFS) chain is located on one lattice site, the lattice spacing is naturally equal to the bond length of $b_0 = 1.0$ nm. Thus, the system size corresponds to the real system of $40 \times 40 \times 250\ nm^3$. The total chain number is set as $n = 300$ with each chain length of $N = 200$. The other lattice sites are occupied by the good solvents and air molecules, with the attractive and repulsive interactions to the chain monomers. This model coupled with a evaporation process will generate a polymer thin films with given monomer densities.

The motion of polymer chains is generated through a micro-relaxation model [7,8], which allows each segment to change positions with its neighboring solvent sites, accompanied by the sliding diffusion along the chain direction if necessary. Conventional metropolis sampling was employed in each micro-relaxation step with the potential energy change:

$$\frac{\Delta E}{k_B T} = \left(b_0 \frac{B_{mf}}{E_c} + b_1 \frac{B_{ma}}{E_c} + b_2 \frac{B_2}{E_c} + b_3 \frac{B_3}{E_c} + c\right) \frac{E_c}{k_B T} \qquad \text{(Supplementary Eq. 3)}$$

Here E_c is the bending energy for two adjacent bonds connected along the chain and c is the total number of non-collinear connected bond pairs along the chain. k_B is the Boltzmann constant and T

is the temperature. The factors b_0 to b_3 show the total number of contacting neighbors. Here $\frac{B_{mf}}{E_c} = \frac{B_{ma}}{E_c} = 0.5$ correspond to the pair repulsion between PMMA-PFS monomers and PMMA-air molecules, $\frac{B_{sa}}{E_c} = 0.5$ refers to the pair repulsion between solvent-air molecules. We choose $\frac{B_{fa}}{E_c} = -0.5$ to denote the attraction between PFS-air molecules, which tends to diffuse the PFS monomers towards the free surface, generating the film surface properties similar to experimental observations. We consider the interactions between PMMA-solvent as 0, indicating the athermal solvent condition.

In our simulation, polymer chains are randomly generated within the box with the density of 0.2. Subsequently the good solvents were evaporated to generate a bulk polymer density of 0.8 and relaxted at reduced temperature $T_r = k_B T / E_c = 3.0$ for 20000 MC steps. Then we heat the film to 10.0 for 10000 MC steps, then lower the temperature to 3.0 again, eventually we obtain the final state of P(MMA-*sta*-PFS) thin films. Then we equilibrate thin films at this density for 100000 steps to get the average local monomber mobility, which is defined in the main text. Such a anealing process will change the surface fraction C_{PFS}, and see the details in the next section.

7. Validation of the formation of loop structure by simulations

The surface fraction of PFS at the film surface (C_{PFS}) can be calculated via the following formula from Monte Carlo simulations:

$$C_{\mathrm{PFS}} = \langle \frac{N_{\mathrm{PFS},surface}}{N_{t,surface}} \rangle \qquad \text{(Supplementary Eq. 4)}$$

here $N_{\mathrm{PFS},surface}$ and $N_{t,surface}$ are the number of PFS and total monomers at the surface layer in our simulations. The $\langle \cdots \rangle$ indicates the statistical average. The blue lines and points in Supplementary Figure 5 shows the measured surface C_{PFS} on the surface of the film at the temperature $T_r = 3.0$ and the weight fraction of random copolymer $X_c = 0.17$. Subsequently, we heating the film to $T_r = 10.0$ for 50000 MC steps, and then lower the temperature to $T_r = 3.0$. Subsequently we measure the surface C_{PFS}, and plot the results as the orange symbols in Supplementary Figure 5. One can clearly see that the increase of the areal fraction of PFS at surface after annealing, predicted by Monte Carlo simulations is also in line with increment trend of surface F/C ratio and water contact angle shown in Supplementary Figures 6 and 7, respectively. Thus, the tendency of surface enrichment of PFS and concomitant surface depletion of MMA reinforces the notion of loop formation at the air interface. More than 85% surface chains forms into loop conformation at the surface of films with various f_{PFS} (Supplementary Table 3).

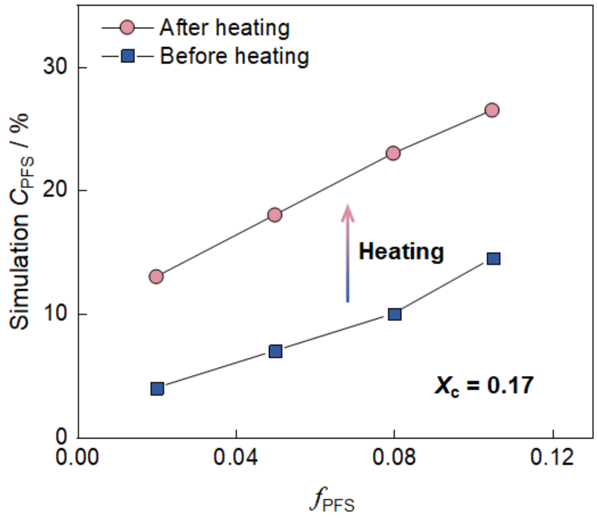

Supplementary Figure 5. The surface segregation of PFS units in the simulated films. The areal fraction of PFS (C_{PFS}) at surface of PMMA/P(MMA-*sta*-PFS) films with various f_{PFS} measured from Monte Carlo simulations at $T_r = 3.0$ before and after annealing the film to $T_r = 10.0$. The weight fraction of the random copolymer is X_c=0.17.

Supplementary Table 3. The fraction of surface chains forming loops at the surface

f_{PFS}	fraction of surface chains forming loops (%)
0.02	85.1
0.05	86.1
0.08	87.7
0.0105	85.9

8. Experimental validation of the increase of concentration of surface loops with the weight fractions of P(MMA-*sta*-PFS) in the films

We used the X-ray photoelectron spectroscopy (XPS) and water contact angle measurement to characterize the changes of the PFS content and concentration of loops at the surface. As shown in the Supplementary Figure 6, the surface F/C atomic ratios at surface increase with weight fraction of P(MMA-*sta*-PFS) in the films (X_c). Data sets in Supplementary Figure 7 show the increase of water contact angle with X_c due to surface segregation of PFS. The areal fraction of PFS at the film surface (C_{PFS}) can be estimated from the water contact angle via Cassie's equation which links the surface wettability of a compositional heterogeneous surface to the relative contents of each component [12]:

$$\cos\theta = C_{PFS}\cos\theta_{PFS} + C_{MMA}\cos\theta_{MMA} \quad (C_{PFS} + C_{MMA} = 1) \qquad \text{(Supplementary Eq. 5)}$$

θ, θ_{PFS} and θ_{MMA} are static contact angles of water for the PMMA/P(MMA-*sta*-PFS), PMMA and PPFS films, respectively; C_{MMA} represents the relatively content of MMA on the film surfaces. Figure 1C in the main text shows the detail trend of increment of C_{PFS} with X_c.

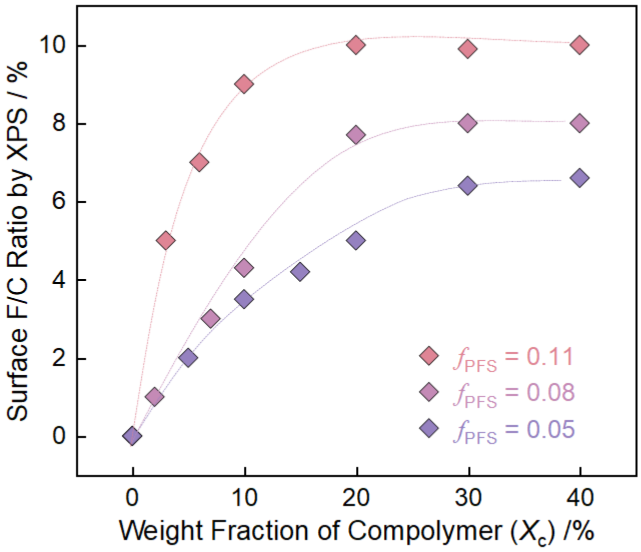

Supplementary Figure 6. Chemical composition at the surface of blended films measured by XPS. Atomic ratio of fluorine and carbon (F/C) at surface of the PMMA/P(MMA-*sta*-PFS) films. The F/C ratios were determined by XPS with a PHI5000C ESCA System with an Mg Kα X-ray source (1253.6 eV) at a take-off angle of 15° corresponding to a detection depth of ~ 2.3 nm [13]. The dashed line was a guidance for the eye.

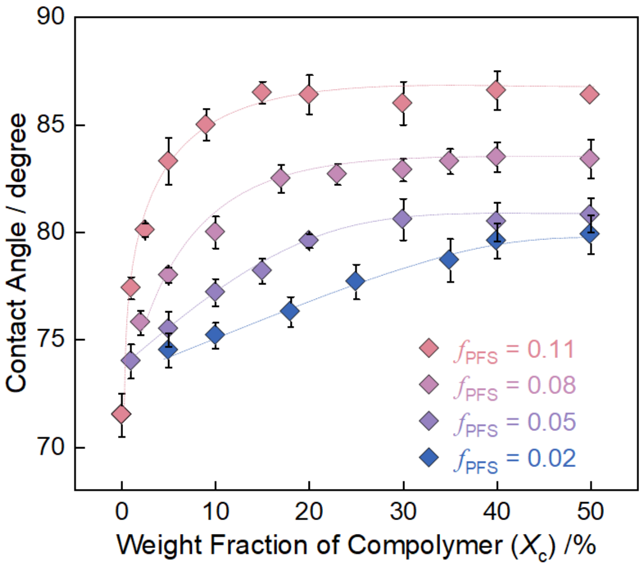

Supplementary Figure 7. Surface water contact angle of blended films. Water contact angle of blended films of PMMA/P(MMA-*sta*-PFS) as functions of X_c. Error bars are ± standard errors.

9. Study of the aggregation behavior of P(MMA-*sta*-PFS) in PMMA matrix

We used small angle X-ray scattering (SAXS) to check if P(MMA-*sta*-PFS) forms any kinds of aggregates in PMMA matrix, Supplementary Figure 8. The measurement was carried out on a SAXS/WAXS system (Xeuss, Xenocs, France) equipped with Cu Kα radiation at an incident X-ray wavelength of 1.6 Å; the scattering signal was integrated along the Debye–Scherrer ring using Rigaku model R-AXIS Display software, affording a one-dimensional scattering profile. Prior to measurement, the samples were equilibrated at 150 °C under vacuum for 24 h. The P(MMA-*sta*-PFS)/PMMA blends with different f_{PFS} and X_c show similar SAXS profiles to that of pure PMMA with no obvious diffraction peak, indicating the absence of aggregation or phase separation of PFS in the PMMA matrix.

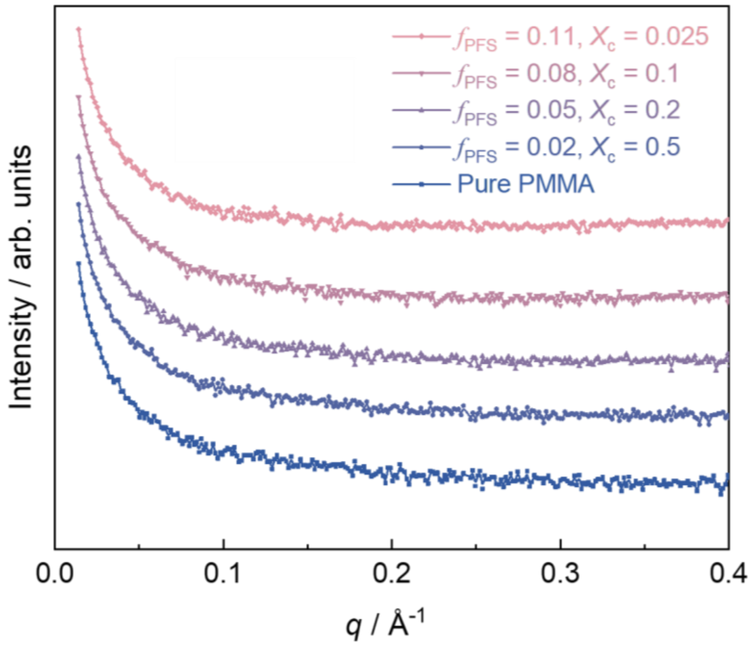

Supplementary Figure 8. The phase structure of the blend films. The SAXS profiles of P(MMA-*sta*-PFS) in PMMA matrix with various f_{PFS} and X_c.

10. Surface loss tangent imaging

Viscoelastic dissipation of the film surface was measured by the amplitude modulation-frequency modulation AFM. The measurement was conducted using an Oxford MFP-3D Origin+ AFM in the loss tangent mode at 297 K and 343 K. Silicon cantilevers with curvature radius of 15 nm and nominal spring constant of 40 N/m (NT-B20-v0010, Nanotool) were used. The cantilever was driven vibrating near its resonant frequency (~330 kHz) to scan across the top surfaces of samples. Surface height and phase shift (δ) of the cantilever oscillation from the driving force was simultaneously recorded during the scanning. The surface height image represents the fluctuation of surface topography, and the loss tangent (tanδ) image corresponds to the spatial distribution of the dissipated energy during the periodic tip-sample interaction. The free amplitude, amplitude ratio and scan rate were set to be identical for viscoelastic measurements of all the film surfaces.

11. Loss tangent of bulk PMMA

The loss tangent of bulk PMMA was measured by dynamic mechanical analysis (DMA) test (DMA1, Mettler Toledo) using the temperature-sweep mode at a series of frequencies (1 Hz < f < 20 Hz), see Supplementary Figure 9A. The tanδ value in the glassy state is about 0.1, and this value gradually increases with temperature in the sub-T_g region, reaching ~ 0.7 at T_g; it then decays to ~ 0.1 at the rubbery state. Assuming the shape of the tanδ ~ T curve was invariant with frequencies, the temperature spectra of tanδ was extrapolated to wider range of f using the VFT relation between f and T (Supplementary Eq.6) for bulk PMMA [14], see Supplementary Figure 9B.

$$\frac{1}{f} = \frac{1}{f_0}\exp\left(\frac{B}{T - T_\infty}\right) \qquad \text{(Supplementary Eq. 6)}$$

A bulk T_g of 452 K is estimated at f = 330 kHz, which is more than 100 K higher than the experiment temperatures for surface loss tangent imaging (T = 293 K and 343 K), revealing the deep glassy state of the underlying PMMA bulk. As the surface T_g is approximately 40 ~ 60 K lower than bulk T_g [15], it's inferred that the T_g at surface of PMMA at measurement frequency of 330 kHz is no less than 350 K. Accordingly, the surface of PMMA at 298 and 343 K are in the glassy or sub-T_g region, where the tanδ increases with temperature as well as the segmental mobility, see Supplementary Figure 9. Moreover, an increase of surface tanδ from 0.27 to 0.51 with increasing the temperature (i.e., increasing segmental mobility) from 298 to 343 K, as shown in Figure 2, further confirmed the positive correlations between surface tanδ and the surface mobility.

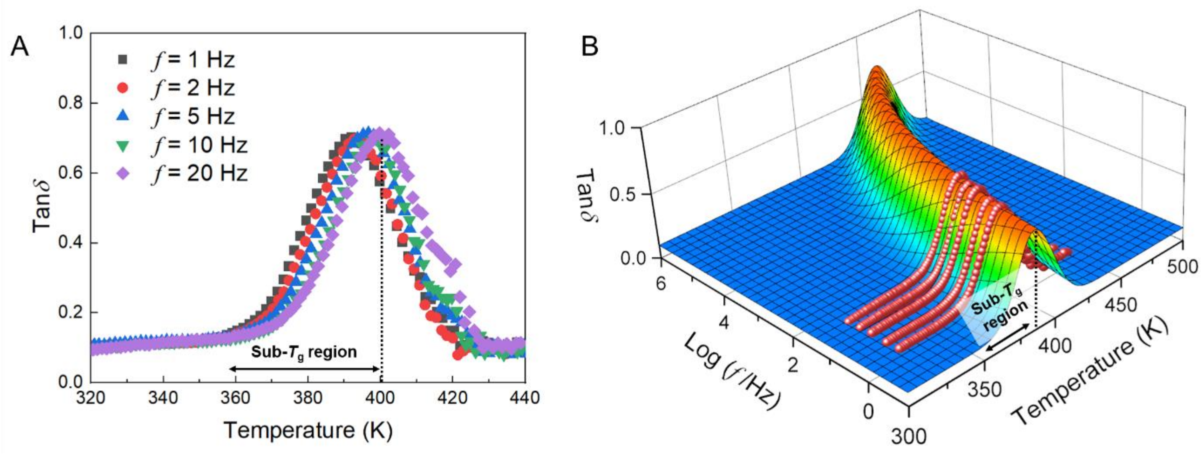

Supplementary Figure 9. The loss tangent of bulk PMMA measured by DMA. (A) The tanδ ~ T curve measured by DMA at different frequencies. (B) The 3D plot of tanδ as a function of temperature and frequency, the red ball represents the tanδ values in panel A. Assuming a frequency independent shape of the tanδ ~ T curve, the 3D plot of tanδ was constructed by extrapolating the data in panel A to wider frequency range on the basis of the VFT relation between time (f^{-1}) and temperature (T) for PMMA [14].

12. Surface loss tangent image of PMMA/P(MMA-*sta*-PFS) blend films

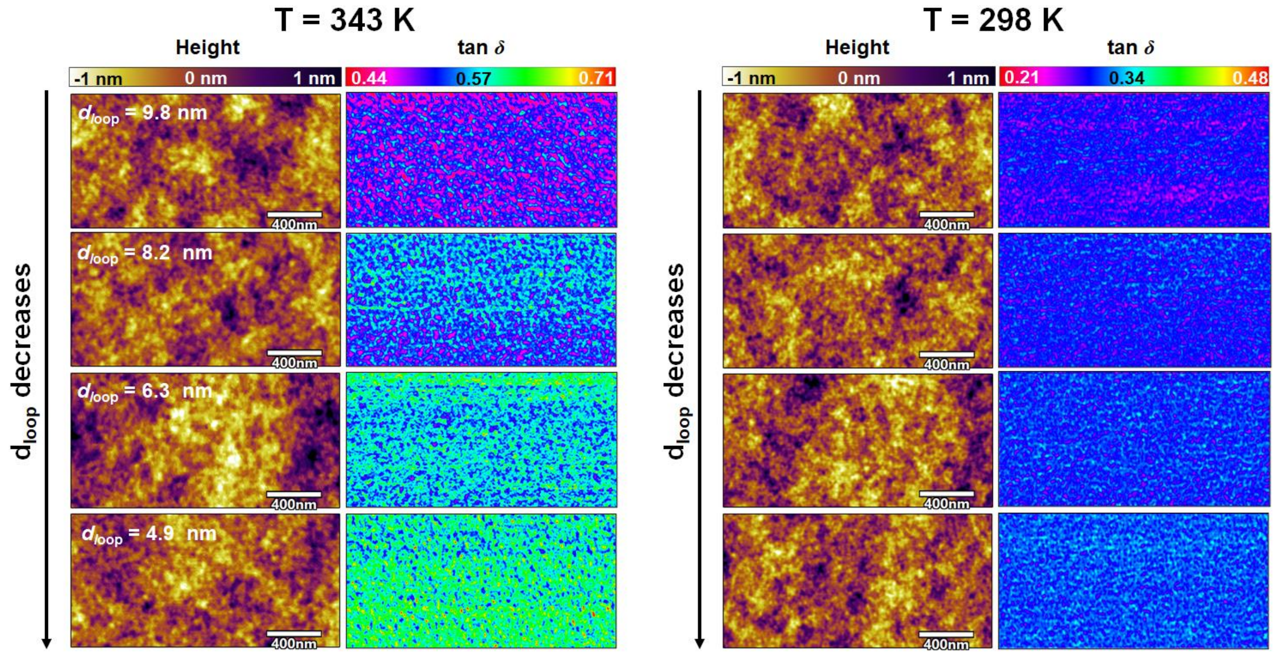

Supplementary Figure 10. Surface loss tangent and morphology of blended films measured by AFM. Surface topographic and loss tangent images of PMMA/P(MMA-*sta*-PFS) films with various f_{PFS} and d_{loop} at 298 and 343 K. As shown in the topographic images, the film surfaces were smooth with an RMS roughness of < 0.5 nm, indicating a uniform distribution of PFS at the surface without phase separation. Thickness of the films is 300 nm.

13. Measurement of glass transition temperature

The glass transition of thin films was probed using a variable-temperature EP4 SW auto-nulling ellipsometer (Accurion Co.; Germany) at a fixed incident angle of 60° with a wavelength of 658 nm. The phase difference, Δ(T) and amplified, ψ(T), from which the film thickness was calculated, were recorded as a function of temperature at a heating ramp of 2 K/min. The T_g of thin films was obtained by fitting the temperature dependence of film thickness to Supplementary Eq.7 [16].

$$h(T) = w\left(\frac{M-G}{2}\right)\ln\left(cosh\left(\frac{T-T_g}{w}\right)\right) + \left(T-T_g\right)\left(\frac{M+G}{2}\right) + c \qquad \text{(Supplementary Eq. 7)}$$

Here, w is the width of the transition zone, M and G are the slope of the liquid line and the glass line respectively, c is the film thickness at T_g. Supplementary Figure 11 shows a typical example of determining the T_g of thin films from the temperature dependence of film thickness obtained by ellipsometry.

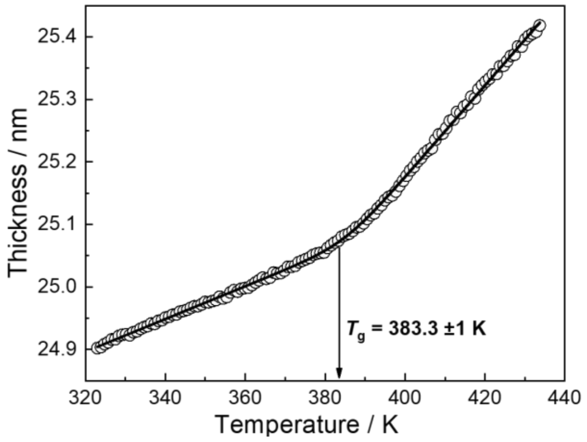

Supplementary Figure 11. The determination of T_g of thin films by ellipsometry. Temperature dependence of film thickness of 25-nanometer-thick blended films on SiOx substrate with f_{PFS}= 0.064 and X_c = 15 %. The black curves fitting results of the data using Supplementary Eq.7.

Supplementary Table 4. The values of A and standard errors

d_{loop} (nm)	A	
	SiO_x substrate	Si-H substrate
10.3	-0.37 ± 0.04	-0.02 ± 0.007
9.8	-0.20 ± 0.04	0.07 ± 0.007
8.2	-0.15 ± 0.02	0.13 ± 0.01
6.3	0.22 ± 0.03	0.33 ± 0.02
4.9	0.26 ± 0.03	0.49 ± 0.04

References

[1] D. Owens and R. Wendt, Estimation of the surface free energy of polymers, Int. J. Appl. Polym. Sci **13**, 1741-1747 (1969).

[2] T. L. Bucholz and Y. L. Loo, Phase behavior of near-monodisperse semifluorinated diblock copolymers by atom transfer radical polymerization, Macromolecules **39**, 6075-6080 (2006).

[3] B. Zuo, C. Li, Y. Li, W. Qian, X. Ye, L. Zhang, and X. Wang, Toward achieving highly ordered fluorinated surfaces of spin-coated polymer thin films by optimizing the air/liquid interfacial structure of the casting solutions, Langmuir **34**, 3993-4003 (2018).

[4] M. Fineman and S. D. Ross, Linear method for determining monomer reactivity ratios in copolymerization, J. Polym. Sci. **5**, 259-262 (1950).

[5] W. A. Pryor and T. L. Huang, The kinetics of the polymerization of pentafluorostyrene, Macromolecules **2**, 70-77 (1969).

[6] B. Zuo, H. Tian, Y. Liang, H. Xu, W. Zhang, L. Zhang, and X. Wang, Probing the rheological properties of supported thin polystyrene films by investigating the growth dynamics of wetting ridges, Soft Matter **12**, 6120-6131 (2016).

[7] Q. Tang, W. Hu, and S. Napolitano, Slowing down of accelerated structural relaxation in ultrathin polymer films, Phys. Rev. Lett. **112**, 148306 (2014).

[8] Q. Tang, M. Müller, C. Y. Li, and W. Hu, Anomalous ostwald ripening enables 2D polymer crystals via fast evaporation, Phys. Rev. Lett. **123**, 207801 (2019).

[9] P. G. de Gennes, Reptation of a polymer chain in the presence of fixed obstacles, J. Chem. Phys. **55**, 572–579 (1971).

[10] P. G. de Gennes, Glass transitions in thin polymer films. Eur. Phys. J. E **2**, 201-205 (2000).

[11] P. G. de Gennes, Glass transitions of freely suspended polymer films, C. R. Acad. Sci. Paris, Ser. IV **1**, 1179-1186 (2000).

[12] A. B. D. Cassie, Contact angles, Discuss. Faraday Soc. **3**, 11-16 (1948).

[13] B. Zuo, Y. Liu, L. Wang, Y. Zhu, Y. Wang, and X. Wang, Depth profile of the segmental dynamics at a poly(methyl methacrylate) film surface, Soft Matter **9**, 9376-9384 (2013).
[14] D. Prevosto, M. Lucchesi, S. Capaccioli, R. Casalini, and P. A. Rolla, Correlation between configurational entropy and structural relaxation time in glass-forming liquids, Phys. Rev. B **67**, 174202 (2003).
[15] T. Kajiyama, K. Tanaka, and A. Takahara, Study of the surface glass transition behaviour of amorphous polymer film by scanning-force microscopy and surface spectroscopy, Polymer 39, 4665 (1998).
[16] K. Dalnoki-Veress, J. A. Forrest, C. Murray, C. Gigault, and J. R. Dutcher, Molecular weight dependence of reductions in the glass transition temperature of thin, freely standing polymer films, Phys. Rev. E **63**, 031801 (2001).
